# Double layer effects in a model of proton discharge on charged electrodes

**DOI:** 10.3762/bjnano.5.111

**Published:** 2014-07-07

**Authors:** Johannes Wiebe, Eckhard Spohr

**Affiliations:** 1 Faculty of Chemistry, Duisburg-Essen University, D-45117 Essen, Germany; 2 Faculty of Chemistry and Center for Computational Sciences and Simulation (CCSS), Universität Duisburg-Essen, D-45117 Essen, Germany

**Keywords:** electrocatalysis, interfacial electrochemistry, proton discharge, reactive force field, trajectory calculations

## Abstract

We report first results on double layer effects on proton discharge reactions from aqueous solutions to charged platinum electrodes. We have extended a recently developed combined proton transfer/proton discharge model on the basis of empirical valence bond theory to include specifically adsorbed sodium cations and chloride anions. For each of four studied systems 800–1000 trajectories of a discharging proton were integrated by molecular dynamics simulations until discharge occurred. The results show significant influences of ion presence on the *average* behavior of protons prior to the discharge event. Rationalization of the observed behavior cannot be based solely on the electrochemical potential (or surface charge) but needs to resort to the molecular details of the double layer structure.

## Introduction

One of the most fundamental electrochemical reactions is proton discharge from an aqueous solution to a charged electrode, which is the first step of the hydrogen evolution reaction. This basic electrocatalytic reaction and its dependence on the nature and the surface structure of the electrode, on impurities and the electrolyte has been extensively studied for more than 100 years [1]. A theoretical description of the reaction is particularly difficult, since the proton interacts strongly with the aqueous environment. The ability of the proton to attach to single water molecules as a hydronium ion or to larger clusters of molecules such as the so-called Zundel and Eigen cations, H_5_O_2_^+^ and H_9_O_4_^+^, respectively, and their fast interconversion through the Grotthuss hopping mechanism [2–5] opens up myriads of different reaction pathways for this reaction step. It is thus impossible to – even approximately – separate the reactive complex from the environment, which forms the basis of many theoretical treatments of reactivity in the condensed phase.

A microscopic treatment of electrocatalytic reactivity needs to accommodate the simple facts that (i) there is a multitude of possible reaction pathways in (ii) an ever changing environment that (iii) interacts strongly and ‘chemically’ rather than weakly and physically with the reactive complex at (iv) very different electrostatic environments near electrodes of (v) very different nature, composition and geometry. Recently we started to investigate proton transfer and discharge at charged electrodes on the basis of reactive force field molecular dynamics (MD), which allows us at present to incorporate for a given model the first four of these requirements into a molecular model. As a starting system we chose a simple platinum (111) surface, because experimentally the platinum surface exhibits one of the highest exchange current densities for the proton discharge reaction. Much research effort in electrocatalysis is directed towards replacing this expensive electrocatalyst with cheaper materials and – ideally simultaneously – to further improve the efficiency of the catalyst. In addition, platinum was deemed suitable because substantial simulation work has been done on this system before.

Much work has been done in recent years by using mostly quantum mechanical density functional theory (DFT) to study adsorbate energetics and geometries on many different catalysts and different catalyst surface geometries. In this context water adsorbates and bilayers have been studied extensively. The electrostatic potential has been introduced either through the implementation of sophisticated boundary conditions [6], through balancing of net electrode charges by electrolyte charges [7–8] or through electrostatic reference methods [9–10]. Chen and Sprik [11] have recently reviewed the current state of such approaches in the context of electronic energy level alignment.

The large number of possible proton transfer paths in the fluxional hydrogen bonding network of the aqueous solution makes the use of quantum chemistry-based approaches difficult but possible. The approach has been pushed forward successfully by the Otani group [12–15], but is limited to the study of few trajectories due to the huge computer time requirements. We chose instead a reactive force field procedure to statistically study the large number of proton transfer pathways by developing empirical valence bond (EVB) force fields for Grotthuss style proton migration and proton discharge at the water/Pt(111) [16–17] and the water/Ag(111) interface [18]. The first EVB models were developed by Warshel to study proton transfer mechanisms in biological systems [19–21]. This methodology was later extended by various groups to study proton dynamics in water [22–23] in a chemically intuitive picture, in which the proton state is described as (to a first approximation) a time-dependent superposition of Eigen, H_9_O_4_^+^, and Zundel, H_5_O_2_^+^, cations. Multistate generalizations of this simple picture were later applied to a variety of physical, chemical and biological problems [24–28]. In order to utilize the methodology for highly acidic environments such as a fuel cell membrane, the approach was, on the other hand, extremely simplified towards a minimal two-state model, in which the proton is either attached to a single water molecule as a H_3_O^+^ ion or to two molecules as a H_5_O_2_^+^ ion [29–30].

The simple two-state EVB model was then combined with a very approximate and qualitative representation of the proton transfer to the surface and the motion of the (discharged) hydrogen atom on the Pt(111) surface. The final MD model can be practically applied in MD simulations of the electrochemical interface. Among other things, it is Hamiltonian in nature and conserves total energy. We have studied in this way proton discharge by straightforward simulation of ensembles of reactive proton trajectories, which all start from a proton equilibrated in the ‘bulk’ (center) of the water slab and migrate towards the charged surface, where they become discharged subsequently. Over the range of surface charge densities that could be simulated, an approximately exponential (or Tafel like) dependence of the microscopically defined rate on the surface charge density was found. Also, comparing a similarly constructed model for the Ag(111) surface showed that the corresponding rates for the silver surface are much smaller than those for the platinum surface. While this seems to be in agreement with the experimental evidence that hydrogen evolution on silver is much slower than on platinum [31–32], it may also be the consequence of model limitations, as both models were constructed in different ways.

In the present manuscript we extend these studies to investigate the influence of ions (Na^+^ and Cl^−^ ions) in the first water layer in contact with the electrode as a first step towards understanding how electrolytes influence proton discharge. In the next section we briefly summarize the details of the simulation procedure. This is followed by the discussion of key results and some concluding remarks.

## Details of the calculations

Our recent publications on a reactive force field model for proton transfer and proton discharge on platinum surfaces on the basis of the empirical valence bond (EVB) approach dealt with idealized water films containing an excess proton on negatively charged platinum and silver surfaces [16–18,33]. Those references describe the models in details, in particular also how parameters for the force field terms were obtained by fitting analytical functions to the data of quantum chemical calculations. The systems were realized as a water film consisting of 512 water molecules plus one excess Zundel complex, H_5_O_2_^+^, in contact with a static platinum slab with (111) surface geometry, consisting of 4 layers with 64 platinum atoms per layer. The surface charge density of the platinum electrode was chosen such that discharge reactions take place on a time scale suitable for MD simulations (within a few tens or hundreds of picoseconds).

Here now we augment the simulated systems by introducing one or two Na^+^ or Cl^−^ ions into the aqueous double layer on the negatively charged electrode surfaces. Specifically, we studied four different systems: double layers with 1 or 2 adsorbed Cl^−^ ions and one with a single adsorbed Na^+^ ion; in addition a reference system consisting of a pure water adsorbate layer was studied. In order to prevent desorption of the negatively charged Cl^−^ ions from the negatively charged platinum surface, the Cl^−^ ions had to be tethered to specific positions on the surface. The tethering of anions does not try to mimic a realistic bonding situation. Rather, it is a simple way to achieve a localized negative charge on the negative surface. Another alternative would have been to keep the position of the anions fixed.

Once adsorbed, the Na^+^ ion, on the other hand, did never desorb from the surface but was free to diffuse within the adsorbate water layer. Hence, while the anions are specifically tethered to a site on the surface, the cation is free to move laterally and, in principle, can desorb. Nevertheless, in the following, we use the term ‘adsorbed’ for both cations and anions.

For each system, 1000 trajectories (only 800 for the system with 1 Cl^−^ ion) were integrated until a time of 2.5 ps *after* the discharge reaction. The platinum surface charge in contact with the pure water film was −5*e*, homogeneously distributed over the area of the slab (*A* = 2.22 × 1.923 nm^2^), which corresponds to a surface charge density of σ = −18.8 μC cm^−2^. Here, *e* = |*e*| is defined as the (positive) absolute value of the electron charge. In the systems with 1 and 2 Cl^−^ ions, each ion carries its full negative charge. The focus of the present work did not reach towards consideration of the electrochemically well-established effect of partial charge transfer, which has been investigated in particular for halogen adsorbates [34]. The magnitude of the homogeneous surface charge was reduced correspondingly by one or two elementary charges *e* so that the total charge of the double layer (homogeneous plus specifically adsorbed charge) was again −5*e*. For the Na^+^ system, the positive elementary charge of the adsorbate ion was not compensated. Instead the homogeneous surface charge was kept at −5*e* so that the total charge of the surface (homogeneous plus specifically adsorbed) amounted to −4*e*.

The water–water, water–hydronium, water–platinum and hydronium–platinum interactions underlying the 9-state EVB model were the same as in the previous work, in which they are described in detail [16]. In the spirit of a maximally simplified model, ion–water and ion–platinum interactions were described by simple Lennard-Jones plus point charge models with ionic Lennard-Jones parameters taken from [35], which were combined with the Lennard-Jones parameters of the water model. Ion–platinum Lennard-Jones parameters were chosen as (ε, σ) = (0.218, 2.93) for Na^+^ and (1.345, 3.35) for Cl^−^, which, together with a harmonic tether potential for the Cl^−^ ions guaranteed that the ions stayed adsorbed in the surface layer of the water molecules. Here, ε is in units of kJ·mol^−1^ and σ in units of nm. In this work, the 9-state EVB model is constructed as the combination of the Walbran and Kornyshev two-state EVB model for proton transport [29] with a model of the hydrogen interaction with the metal surface, which is parametrized by seven distinct EVB basis states. The model describes states in which a proton is bound to water molecules and states in which a (neutralized) hydrogen atom interacts with the surface and superpositions thereof. The proton charge, and particularly its change during the neutralization reaction, is compensated by a corresponding negative charge on the metal slab.

Far from the electrode only the two charged states can contribute to the EVB ground state so that the state of a proton is a time-dependent superposition state of two different H_3_O^+^ states. The proton complex thus dynamically moves between more hydronium and more Zundel like states. In this situation the metal states do not contribute, since the coupling elements to the metal states vanish. After the proton has discharged, its state is a superposition of 7 equivalent hydrogen states in which the atom binds to one of the 7 metal atoms of a hexagonal surface cluster. The number of these states was chosen as the minimal number of hydrogen states that allow for a continuous motion of the hydrogen atom between on-top, hollow, and bridge sites. Shortly before the discharge reaction after the proton has migrated close to the metal surface, the coupling between the protonic and the (discharged) surface states sets in and the full 9-dimensional Hamiltonian matrix is diagonalized. We furthermore make the assumption that the system always stays on the adiabatic ground state potential energy surface, which we obtain as the lowest energy eigenvector of the Hamiltonian matrix. All further details of this model can be found in [16].

In the adiabatic ground state simulations performed here, proton transfer and proton discharge occur mainly when the environment of the proton provides adequate configurations that make the outcome of a proton hop favorable. Thus, the barrier for proton motion is usually small so that proton tunneling is less important than in many other cases. However, there is evidence for quantum effects due to the delocalized nature of the protons, in particular from ab initio MD simulations [4,36–37]. Such calculations show that an adequate incorporation of the wave nature of atoms shifts the (broad) distributions of internal states towards states in which the center of proton charge is more delocalized over two or more water molecules. The two-state EVB model [29] shows indeed a preference for more delocalized (Zundel like) states [38], which is thus a (possibly fortuitous) feature of our model, which on average incorporates some of the quantum effects in an empirical way.

According to estimates we made in [17] the surface charge densities used in our computer simulations falls within the range of hydrogen underpotential deposition (UPD). In particular, at the negative surface charge densities studied here, one can expect the existence of a hydrogen UPD layer and fast discharge, which is indeed consistent with the results of the model. Recent DFT calculations by the Groß group [34] showed that the existence of such a layer moves the water layer to larger distances from the surface and shows a somewhat larger orientational order of the water molecules, which was attributed by the authors to be the result of weakened water–metal interactions in the presence of the hydrogen layer. The differences of the orientational distributions are small, so that we do not expect qualitative differences in the fast reorientation dynamics that accompany the proton discharge step [17]. Thus the overall effect of the presence of the hydrogen layer on the Volmer discharge step should not be large and is furthermore expected to be similar for all studied systems. We have thus chosen not to incorporate the additional complexity of a UPD layer into the model Hamiltonian.

## Results

**Figure 1:**
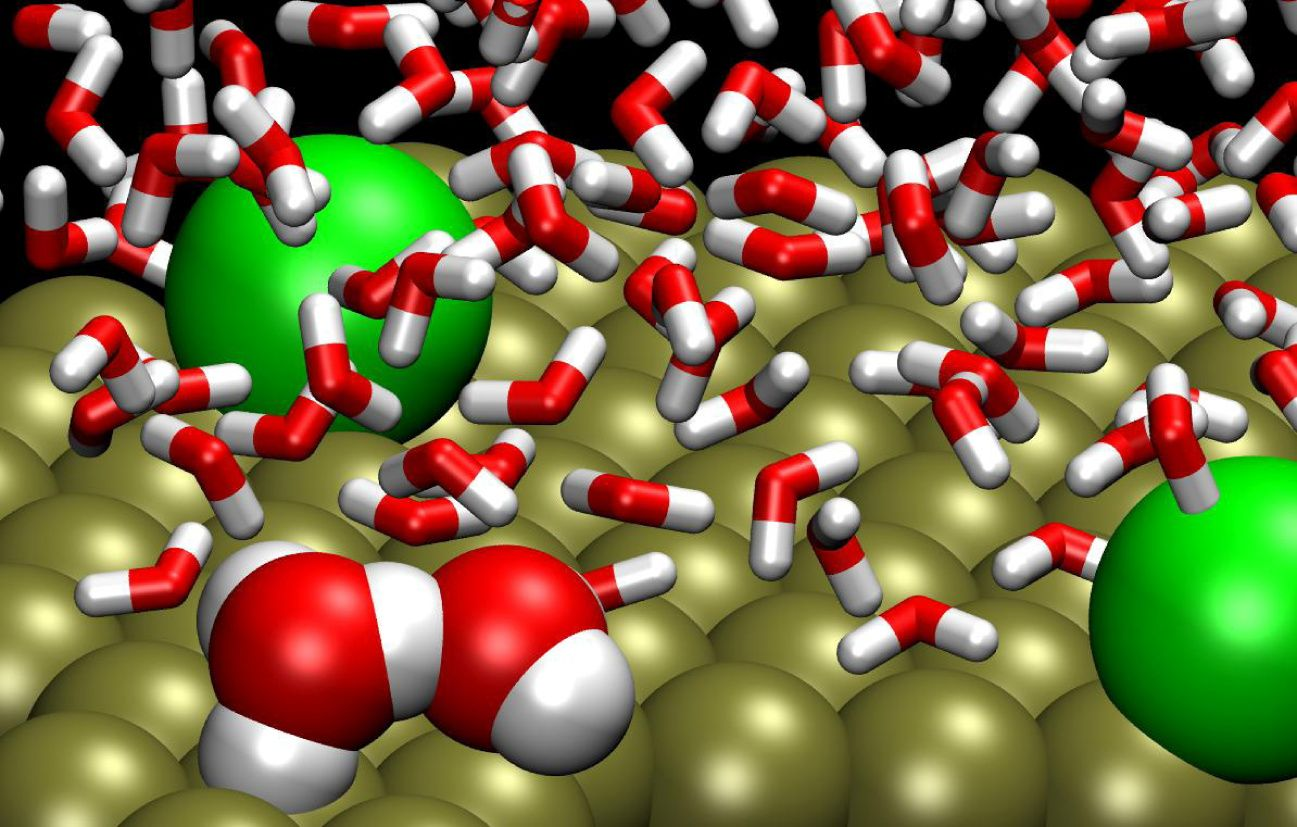
Snapshot for a water film with two adsorbed Cl^−^ ions (green).

Figure 1 shows a system snapshot for the system with two adsorbed chloride ions, about 1 ps before a proton transfer event. The instantaneous Zundel complex is marked as spheres whereas regular water molecules are represented as sticks.

**Figure 2:**
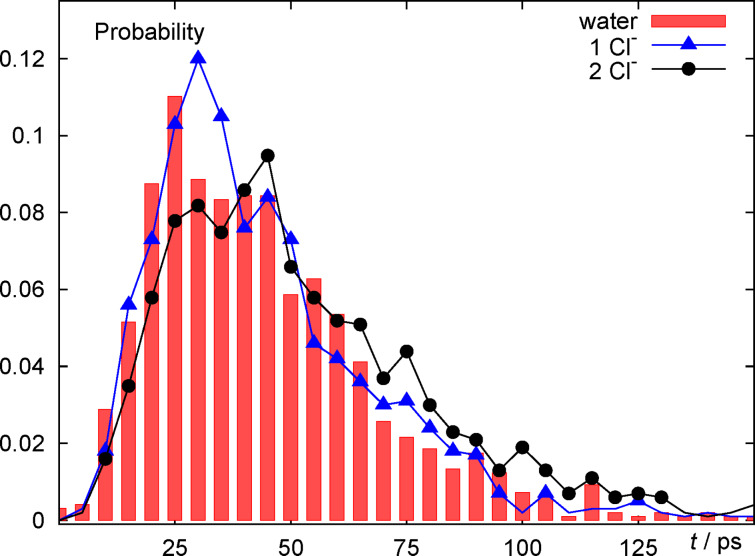
Distribution of discharge times (represented as probabilities to observe discharge within a 5 ps time interval) for a system of pure water (red boxes), of one fully charged adsorbed Cl^−^ ion (blue triangles) and two fully charged adsorbed Cl^−^ ions (black circles). In all three cases the total surface charge was −5*e* and, consequently, the homogeneous surface charge density corresponded to −5*e*, −4*e* and −3*e*, respectively.

The most obvious observable to study with our simulation setup is the time until discharge. The distribution of discharge times is rather broad, which has also been observed for the pure water case [17]. Figure 2 shows the distribution of reaction times for three simulations with a total surface charge of −5*e*. In the pure water simulation, the entire surface charge is homogeneously spread over the metal slab. In the simulations with one (two) contact adsorbed tethered chloride ions, −4*e* (−3*e*) of the slab charge are homogeneously spread over the metal and the remaining one (two) negative charge(s) are centered on the ions. Note that in the present study we have disregarded the fact that the ions, which are adsorbed at the electrochemical interface, usually carry only a partial charge, which is a consequence of the fact that the bond of halide atoms with the metal surface has partially covalent character. Thus, the studied systems represent the idealized case of no partial charge transfer (PCT).

All three distributions are rather similar. Most trajectories react during a time interval of about 25 to 70 ps. However, there is also a trend for the systems with adsorbed chloride to exhibit longer reaction times. This becomes quite apparent, when comparing pure water (red bars) with the 2 Cl^−^ case (black circles); the 1 Cl^−^ case falls in between. Whether or not the slight dependence on ion concentration in the adsorbate layer is due to differences in the average electric fields, which drive proton motion in solution, or the consequence of a site-blocking effect, which might play a role if the proton approaches the negative chloride centers in ‘head-on’ collisions, cannot be decided on the basis of these data (see below).

In the model setup implemented here, in which trajectories start in the center of the water film and protons subsequently migrate towards the charged electrode surface until they become discharged, one can obtain average properties over trajectories in at least two fundamentally different ways: In the first method the trajectories are aligned in a straightforward manner at their starting position. Thus, the average of some observable *O* over trajectories *i*, 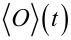 is calculated according to

[1]
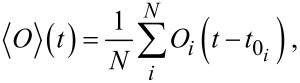


where the sum runs over all trajectories and 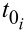 denotes the initial time of the *i*th trajectory run.

**Figure 3:**
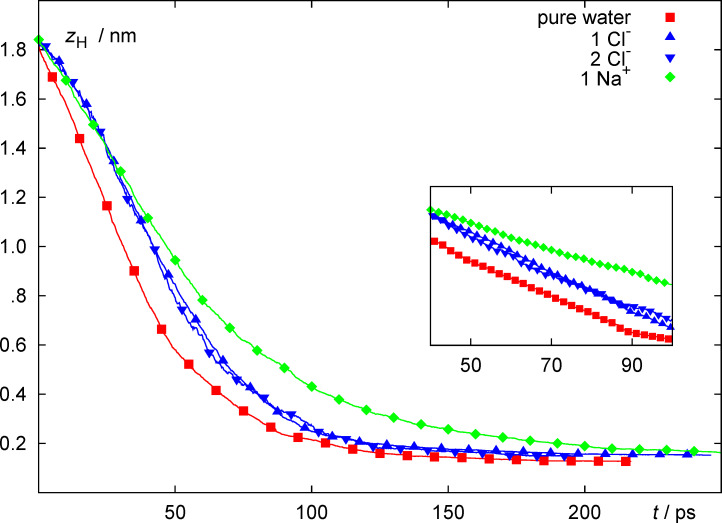
Average proton distance from the metal surface *z*_H_ as a function of time for pure water (red squares), one or two adsorbed Cl^−^ ions (blue triangles) and one adsorbed Na^+^ (green diamonds). All ions carry a full positive or negative charge. The total surface charge is −4*e* for the Na^+^ system and −5*e* for all other systems. Trajectories are aligned and averaged at their starting time. Inset: logarithmic scale.

Figure 3 shows the average distance of the particular proton which, at any given time, is eligible to be transferred to a neighbouring water molecule, and which is ultimately discharged by transfer to the metal surface. After an initial induction period (during which the proton is, on average, accelerated towards the surface) the curve assumes an approximately linear slope in the time interval between about 30 and about 60 ps. This behavior is indicative of the drift regime characteristic for a charged ion migrating in a homogeneous electric field. Thus, proton motion in this regime is dominated by the *mean* electric field (which can be calculated, e.g., by solving Poisson’s equation with the charge density obtained by the average ionic densities), while the instantaneous electric field acting on the proton is fluctuative in nature. Beyond about 60 ps there is an approximately exponential decay of the curve as the inset of the curve shows. This behavior is the consequence of the different possible outcomes of the trajectories in the vicinity of the surface, which exhibit a broad distribution of times, during which the proton carrying complex is adsorbed in the contact water layer but does only dissociate after a configuration suitable for discharge occurs fluctuatively. Note that, in order to avoid excessive noise for long times (when few trajectories contribute to the average, since many trajectories have been terminated already after discharge), we have artificially extended each terminated trajectory by using the constant final value of the transferred proton. With this procedure, the curve must approach a constant value corresponding to the average adsorption distance at infinitely long times. The Na^+^ ion deposited initially on the negatively charged surface did never desorb from the electrode. In the simulations with negative ions one or two Cl^−^ ions were tethered to the surface, since otherwise the ions would have desorbed due to the strong Coulomb repulsion from the homogeneous electrode charge. Note that in the simulations with the anions the overall surface charge was the same as for the pure water case, corresponding to a total of −5 elementary charges (corresponding to a surface charge density of −18.8 μC·cm^−2^). The presence of the Na^+^ ion, on the other hand, reduced the total charge of −5*e* (from the homogeneous part) to a total of −4*e*, corresponding to a total surface charge density of −14.4 μC·cm^−2^.

The general behavior of the curves is similar. However, compared to the pure water case, the induction period seems to be slightly longer for the systems with adsorbed chloride. As could already be inferred from Figure 2, the presence of the chloride ions delays the approach of the proton to the surface relative to the pure water case, in spite of the fact that the total surface charge is identical in all cases. For the Na^+^ simulation the intermediate slope of the curve is the smallest, in line with the fact that the net surface charge density is reduced and thus the driving force for migration and discharge is smaller than in the other cases.

Figure 3 can provide some information about the time scale of the proton approach to the surface and its residence time in the first adsorbed water layer. However, it does not provide any obvious insight into the nature of the discharge reaction. In order to probe the short time behavior immediately before and immediately after the reaction, we have calculated averages over trajectories in a different way: we have aligned the trajectories at their respective time of reaction rather than at the start time. Thus, an alternative definition of a trajectory average

[2]
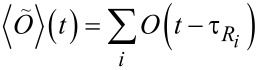


can be used to assume a reaction-centered view of events. 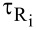 denotes the reaction time of the *i*th trajectory. Thus, comparison is not made based on some (more or less) arbitrary starting time in the past, but around the time of the reaction specific for each trajectory. This is consistent with the picture of chemical reactions as isolated rare events, for which it should suffice to study the behavior of the system from shortly before to shortly after the event. Here, we define the reaction time 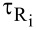 as the time, when the sum of the weights of all metal EVB states is for the first time larger than 0.9 in trajectory *i*, in other words, when more than 90% of the proton charge has been transferred to the surface. In a previous work [17], it was established that once this point has been reached, the discharge process is essentially complete.

**Figure 4:**
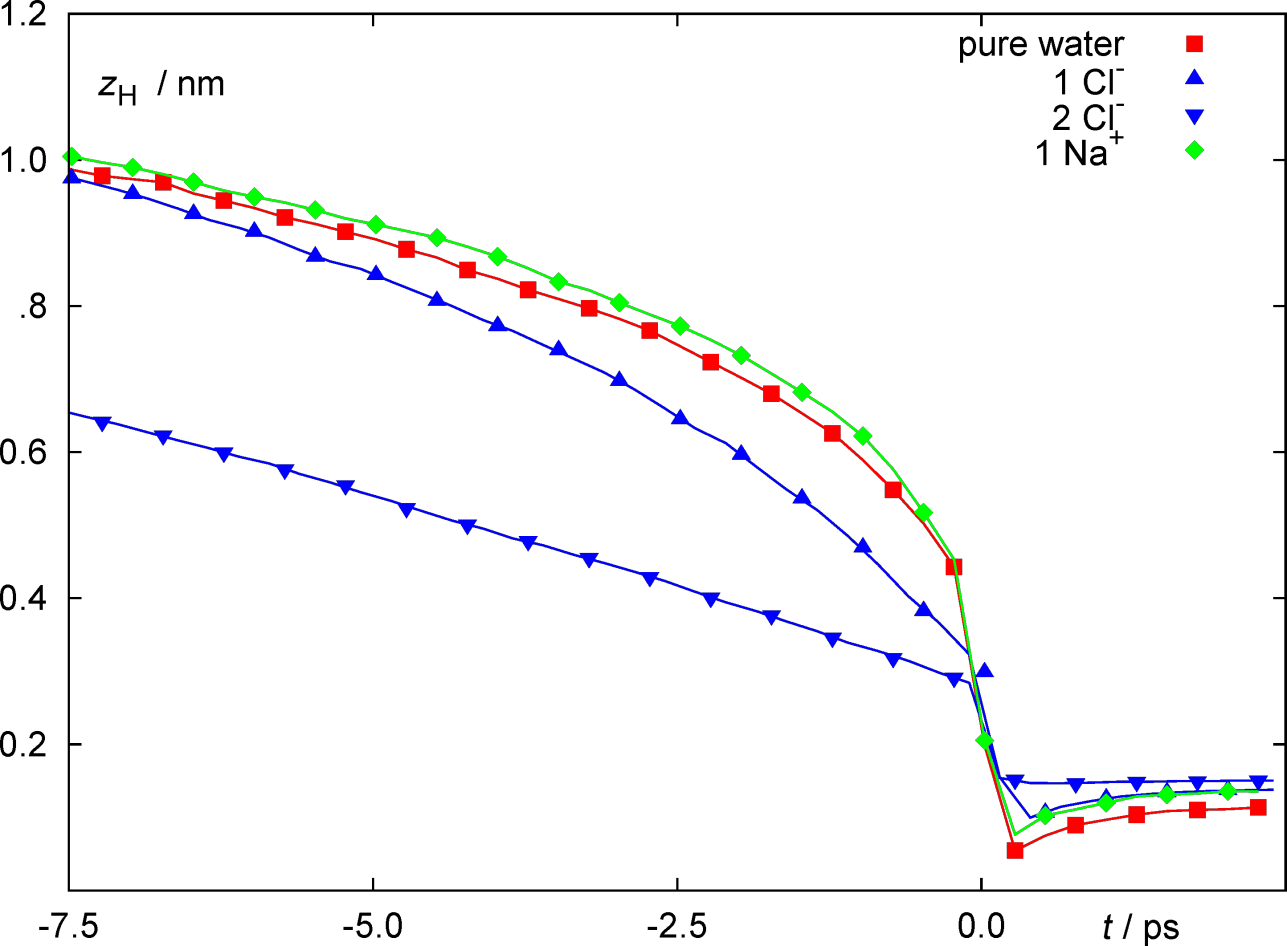
Average proton distance from the metal surface *z*_H_ as a function of time for pure water (red squares), one or two adsorbed Cl^−^ ions (blue triangles) and one adsorbed Na^+^ (green diamonds). All ions carry a full positive or negative charge. The total surface charge is −4*e* for the Na^+^ system and −5*e* for all other systems. Trajectories are aligned at the time of reaction, which is the zero of time, and averaged.

Figure 4 shows the corresponding data for *z*_H_(*t*) for the time interval from −7.5 ps to +2.5 ps, i.e., for the last 10 ps of the reactive trajectories. All trajectories were terminated 2.5 ps after the discharge reaction took place. The curves show significant differences for the different simulated systems. Quite expectedly, all curves have in common that the final step, when the proton moves out of its position as a proton in the first water layer to the state of an adsorbed hydrogen (which is much closer to the surface layer of platinum atoms), is very rapid and occurs within a few femtoseconds, a timespan not resolved in this representation.

If one first focuses on the behavior *after* the reaction, one notes that, due to excessive kinetic energy produced as a consequence of the exothermic discharge reaction, the average proton distance immediately after the reaction is rather small and then relaxes to its equilibrium value further away from the top platinum layer. This behavior is particularly pronounced for pure water and for the Na^+^ simulations. These two simulations also show the fastest final decrease of the average proton distance from the surface in the time interval *before* the reaction takes place. Apparently the proton is accelerated (on average) from a position in the second or in the third layer immediately before the reaction. This supports earlier conclusions that the reaction mechanism at high driving force (i.e., at high surface charge densities) does not require a (fluctuative) reorientation of water molecules in the first layer to take place. Rather, once the proton can be transferred into the adsorbed water layer, proton discharge follows (almost) instantaneously. Although our model does not allow truly simultaneous proton transfer (all proton transfer steps are sequential), the quick succession of several such steps is an indication that such a simultaneous transfer mechanism might be possible in reality.

Interestingly the curves for the pure water simulation and the single Na^+^ ion simulation are very similar before the reaction takes place. Thus, shortly before the reaction the proton motion is not (or only very slightly) affected by the presence of the adsorbed Na^+^ ion. A possible interpretation of this behavior could be the following: the proton is unlikely to be discharged in the immediate vicinity of the positive site, because it is repelled by the positive charge of the Na^+^ ion. Consequently, the proton trajectories most likely ‘bend around’ the contact adsorbed cation. Once the proton is ‘sufficiently’ close to the surface (apparently the second or third layers fulfill this condition already) the proton trajectory is strongly affected by the field of the homogeneous surface charge, while the repulsive field of the Na^+^ ion plays a minor role in bending the proton trajectory away from the Na^+^ ion. Thus, close to the surface, the driving force for proton discharge on the metal electrode with an adsorbed Na^+^ ion is rather similar to the one in the absence of the cation, even though the net electrode charge is different by one elementary charge.

Chloride ions, on the other hand, behave qualitatively different. The simulation with a single Cl^−^ ion clearly shows a much slower approach of the proton to the electrode surface than in the case of the clean surface. The effect is even more significant in the presence of two contact-adsorbed chloride ions. In the latter case the curve shows an almost linear dependence of the average proton distance on time, which indicates that a simultaneous (or, since truly simultaneous transfer is not possible in our model, almost simultaneous) proton transfer from a water molecule in the second layer to one in the first layer and from there to the metal is of minor importance. Note again, that the electrode surfaces in the simulations of the proton with 0, 1, and 2 Cl^−^ ions all carry the same total charge. Thus the difference in behavior shortly before the reaction indicates significantly different local electrostatic potentials and fields. This demonstrates one possible way in which local double layer effects can modify electrochemical reactions in addition to the influence of the external electrode potential.

**Figure 5:**
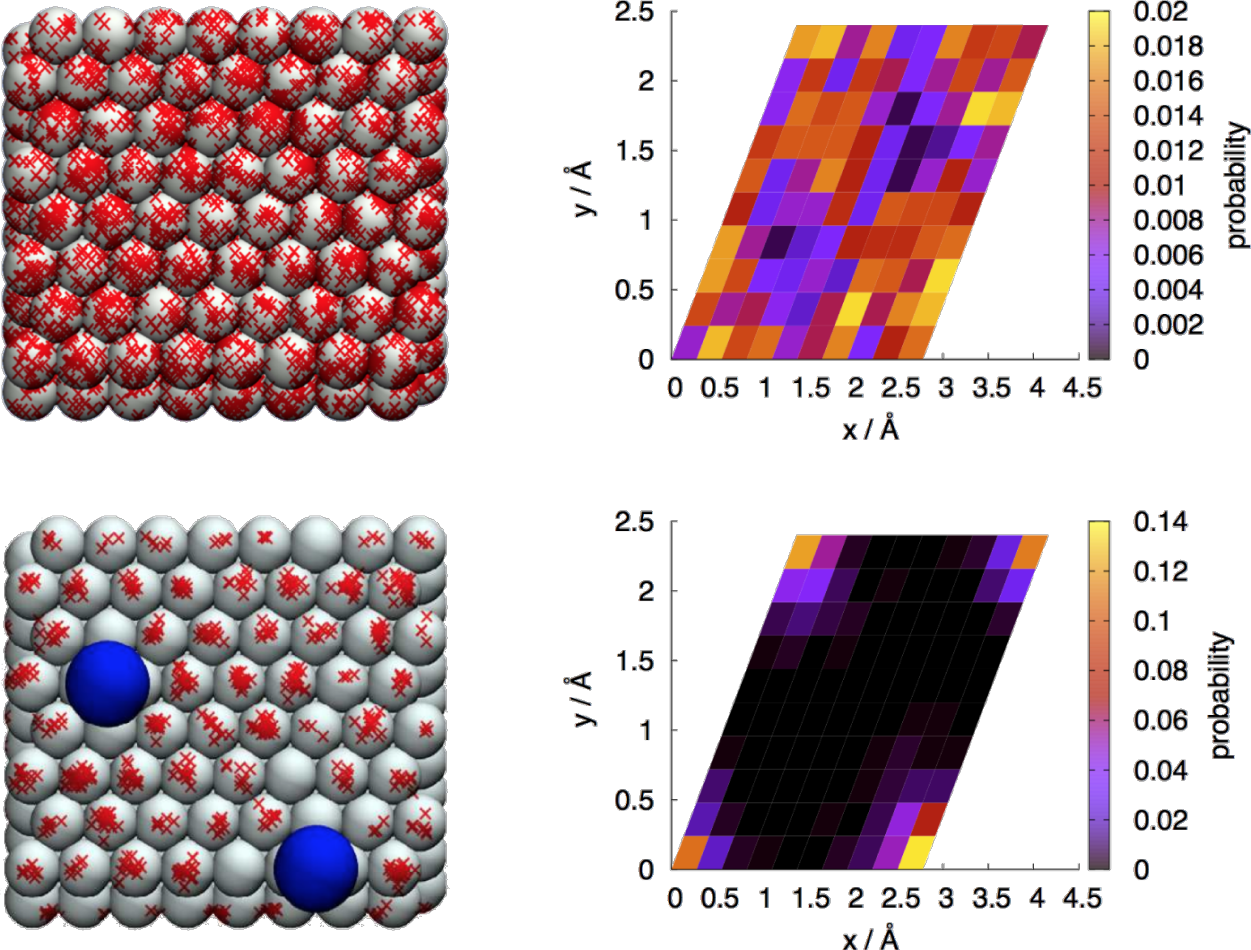
Lateral proton positions at the instant of discharge marked as red × symbols (left) and probability distributions of the proton positions at discharge mapped into a single surface elementary cell (right) for an interfacial layer consisting of pure water (top) and of two solvated Cl^−^ ions (bottom).

Figure 5 shows the lateral positions of the discharging proton above the metal surface as crosses for each individual trajectory on the left at reaction time (time *t* = 0 in Figure 4). The right figure shows the probability density of the proton transfer event as a function of the proton position at discharge *projected* onto the surface and mapped into a surface elementary cell. Note that the potential energy surface of a discharged hydrogen atom on platinum is relatively flat so that the H atom is very mobile. For the pure water case (top) discharge events are observed above all surface sites. However, discharge from straight above individual metal atoms is most probable, because this is the most probable adsorption site for the water molecule and the most probable direction to transfer the proton is along the direction from the oxygen atom to the surface. This behavior is more pronounced in the case of two adsorbed Cl^−^ ions. In this case on-top proton transfer is significantly increased compared to all other adsorption sites. This is, however, in part a consequence of the more ordered water layer structure induced by the two adsorbed ions. The distribution functions within the elementary cell on the right reflect this as well. While for the pure water interface, proton transfer within the elementary cell occurs almost homogeneously everywhere, there is a clear prevalence for on-top sites for the case of two adsorbed Cl^−^ ions.

**Figure 6:**
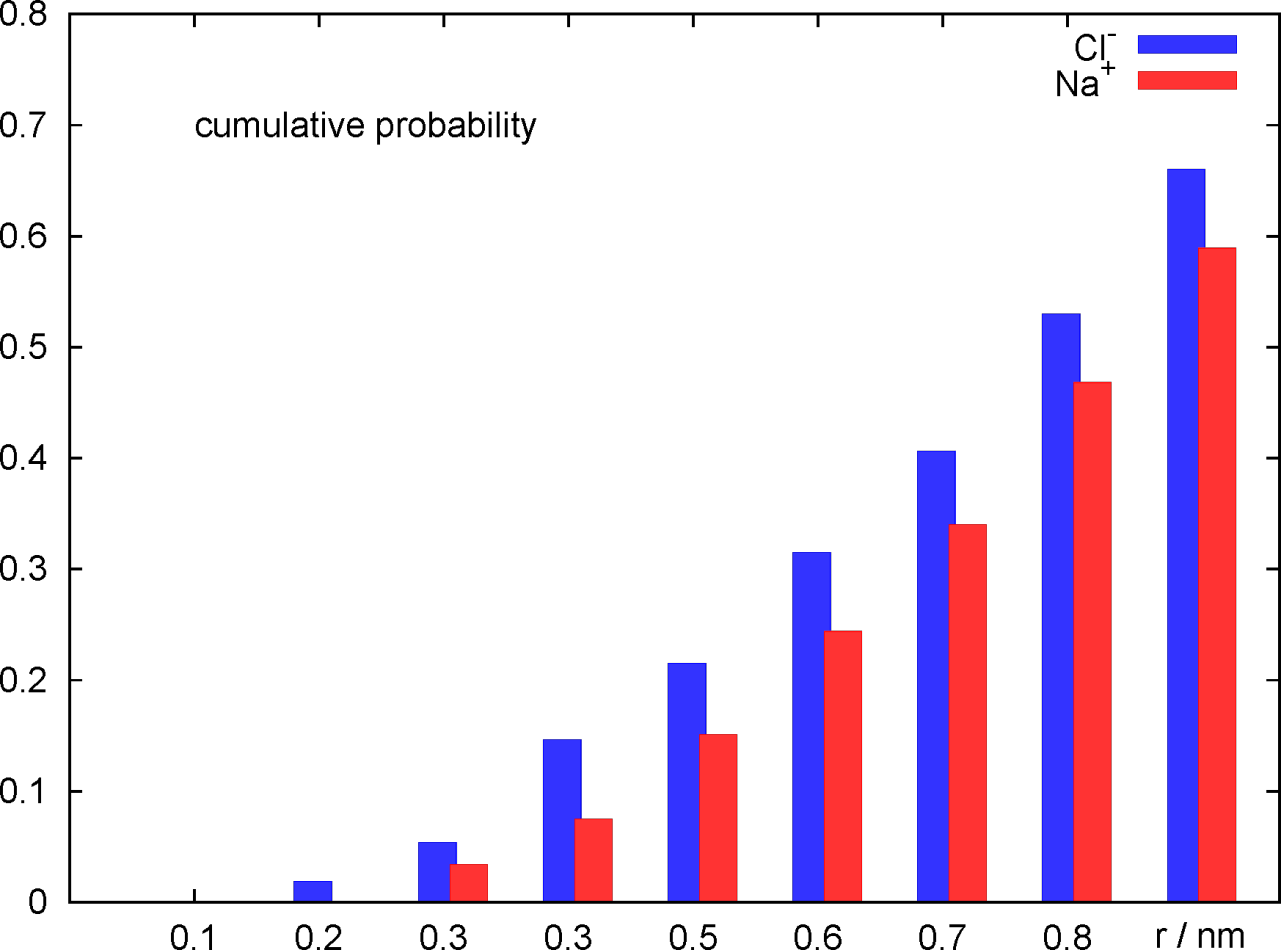
Cumulative probability to observe proton discharge within a radius *r* around the Na^+^ ion (red) and the Cl^−^ ion in the system with only one adsorbed Cl^−^ ion (blue).

Figure 6 shows indeed that there is a preference for the location of proton discharge to be closer to the anion than to the cation. The figure shows, as a function of the radius *r* the cumulative probability for proton discharge to occur within this distance from the adsorbed ion. For all distances shown, the probability is higher around the chloride ion than around the sodium ion, but overall this preference is not very pronounced.

## Discussion

We have compared reactive trajectory calculations of proton discharge from aqueous solutions to charged platinum electrodes for pure water, for adsorbate layers with single Na^+^ and Cl^−^ ions, and for an adsorbate layer with two Cl^−^ ions. All ions have their full positive or negative elementary charge, so that no effects of partial charge transfer to the surface are included in the model. No bulk electrolyte is present that is capable of screening the surface charge. The sole difference between the different systems is the composition of the adsorbate water layer and distribution and magnitude of the negative electrode charge.

For the series of 0, 1 and 2 Cl^−^ ions the overall charge of the combined electrode/adsorbate ion system, which the approaching proton experiences, is identical, because the same total charge was distributed differently between the surface (as a homogeneous surface charge) and the ions (as point charges at the ion site). Nevertheless, significant differences can be observed. The distribution of reaction times, and thus the average, shifts towards higher values with increasing Cl^−^ concentration. Thus, not only the overall long-range electrode potential but also the local charge distribution plays a role for the reactivity. This is probably not so much a consequence of site blocking but rather of enhanced interaction of the proton complex with the negative chloride ions. This interaction manifests itself for instance in Figure 4 where it becomes obvious that the proton approaches the surface much more slowly in the presence of one Cl^−^ ion as compared to water, and even more slowly in the presence of two Cl^−^ ions.

On the other hand, shortly before the discharge step occurs, the presence of the sodium ion plays a minor role, as can be seen from the time dependence of the mean approach distance, which is very similar in the presence of the sodium ion when compared to pure water, in spite of the fact that the total charge of the surface and the adsorbate layer is smaller in the presence of Na^+^. The similarity between the two curves in Figure 4 may be a consequence of the fact that the approaching positively charged proton avoids the positively charged Na^+^ ion. As Figure 6 shows, this effect is indeed present, but it appears to be rather weak. In fact, the proton approaches the surface even slightly faster when the Na^+^ ion is present in the double layer.

The presence of the ionic adsorbates has an ordering effect on the surrounding water molecules (analogous to the one observed by the Gross group for water around an OH group on a Ru surface by using DFT calculations [39]), which is evident from the correlation of the proton discharge sites with the ions and with the on top water site in Figure 5.

In summary our calculations show that, aside from the obvious influence of the interfacial or electrode charge density, structural features of the double layer also have an influence on the electrochemical reactivity, represented here through the proton discharge reaction and its dependence on the composition of the adsorbed water layer. This is an example of a double layer effect which influences the interfacial structure and dynamics beyond the simple one-dimensional potential drop at the interface.
